# Checking facts and fighting back: Why journalists should defend their profession

**DOI:** 10.1371/journal.pone.0208600

**Published:** 2018-12-10

**Authors:** Raymond J. Pingree, Brian Watson, Mingxiao Sui, Kathleen Searles, Nathan P. Kalmoe, Joshua P. Darr, Martina Santia, Kirill Bryanov

**Affiliations:** 1 Mass Communication, Louisiana State University, Baton Rouge, Louisiana, United States of America; 2 Media and Communication, Ferrum College, Ferrum, Virginia, United States of America; 3 Mass Communication and Political Science, Louisiana State University, Baton Rouge, Louisiana, United States of America; The University of Hong Kong, HONG KONG

## Abstract

Bias accusations have eroded trust in journalism to impartially check facts. Traditionally journalists have avoided responding to such accusations, resulting in an imbalanced flow of arguments about the news media. This study tests what would happen if journalists spoke up more in defense of their profession, while simultaneously also testing effects of doing more fact checking. A five-day field experiment manipulated whether an online news portal included fact check stories and opinion pieces defending journalism. Fact checking was beneficial in terms of three democratically desirable outcomes–media trust, epistemic political efficacy, and future news use intent–only when defense of journalism stories were also present. No partisan differences were found in effects: Republicans, Democrats, and Independents were all affected alike. These results have important implications for journalistic practice as well as for theories and methods of news effects.

## Introduction

Fact checking is central to the role of journalism in a healthy democracy, whether it occurs in explicitly labeled fact check segments or within ordinary stories [1][2][3]. In theory it can correct misperceptions and create reputational costs for elites who make false claims. However, it may also be the media who incur reputational costs for fact checking. Fact checking has been found to sometimes fail or even backfire in its effects on factual beliefs [4][5][6]. Such negative effects may extend to trust in and use of news if people see fact checking as a sign of bias. Alternatively, fact checking might have positive effects if people see it as a sign of journalists pursuing truth and holding elites accountable, or if they can somehow be persuaded to see it this way.

One possible remedy is for journalists themselves to speak up in defense of their profession. A rising tide of anti-media rhetoric has renewed debate among journalists about how to respond or whether to respond at all. Traditionally, many journalists have argued that responding would violate norms of neutrality, and instead claim that just doing good journalism will be enough to demonstrate that anti-media rhetoric is incorrect [7]. The latter is an empirical claim, and one that we are very skeptical of in light of past research [8][9][10][11]. We argue that defense of journalism could help audiences appreciate good journalism generally, and fact checking specifically.

We propose that the combination of fact checking and defense of journalism could help restore trust in and use of news media, while also restoring confidence in the existence and attainability of facts in politics. We assess the latter possibility using epistemic political efficacy (EPE), which is self-confidence in an ability to figure out whether factual political claims are accurate [12]. Experiments reviewed below have found fact-checking can affect EPE as well as attitudes toward media, but have not simultaneously tested effects of defense of journalism, and have all relied on assigning people to read stories they may not have chosen on their own. Choice is rarely incorporated into media effects experiments, but can dramatically alter results [13].

This study uses a field experiment embedded in an online news portal to test effects of fact checking and defense of journalism on media trust, EPE, and future news use intent. Participants were paid to spend five days using a purpose-built news portal containing many real, timely news stories and a few experimental treatment stories. These treatment stories were also real news stories, and were identified as either fact check stories or defense of journalism using a combination of keyword searches and researcher verification. This innovative method allows a causal test of effects of fact checking and defense of journalism during choice-driven use of real online news.

### Attacking and defending journalism

When faced with unfavorable news coverage, politicians of all stripes attack media as a strategy to avoid accountability [11]. In the U.S. context, claims of liberal media bias are far more common, and rose dramatically in the 1980s and 1990s [14], after becoming a staple of conservative rhetoric in the Nixon era [15]. This was facilitated further by the rise of various forms of opinion-based alternatives to mainstream news, including conservative talk radio in the 1990s, followed by partisan cable news and then online partisan opinion sites [16][17][18]. Recently this rhetoric has become alarmingly extreme, led by the unprecedented example of a U.S. President regularly accusing mainstream reporters of making up anonymous sources and doing “fake news” [19], and even calling the press the “enemy of the American people” [20]. A very mild and reasonable version of this bias critique is implicit in the original meaning of the term “bias:” concern that journalists have a tendency to *unintentionally* favor Democrats despite their efforts to be impartial. Somewhere along the way, arguably with roots long before the current administration [21], this rhetoric transformed into a conspiracy theory in which mainstream journalists are seen as partisan actors writing intentionally misleading or even entirely fake stories.

Some journalists have responded in defense of the crucial role of the press in democracy as a check on elite dishonesty and a custodian of facts. For example, Carl Bernstein pulled no punches in response to Trump’s first tweet referring to the press as the “enemy of the American people,” saying “Donald Trump is demonstrating an authoritarian attitude and inclination that shows no understanding of the role of the free press” [20]. Other journalists, like ProPublica reporter Jessica Huseman, see engagement with anti-media rhetoric as fruitless, concluding “Save your straight-to-camera monologues, and spend that time accomplishing journalism that history will bear out” [7]. Similarly, Washington Post media columnist Margaret Sullivan concluded a piece about anti-media rhetoric with “It will be journalists’ continued challenge not to take the bait, to refuse to play the assigned role of presidential enemy” [19], and in another piece asserted “Most journalists—among them the very best—believe that if they keep presenting the facts and countering the spin that that will be enough” [22]. More recently, the tide seems to have turned in the long-running debate among journalists about whether to respond to attacks, as evidenced by The Boston Globe’s August 2018 campaign in which 300 newspapers published editorials defending journalism [23].

Research on the effects of anti-media rhetoric suggests ignoring it is ill advised. Accusations of media bias by elites from either party have strongly reduced media trust in experimental trials [9][10][11]. Exposure to conservative talk radio, a medium rich in anti-media rhetoric, is a strong predictor of low media trust [16]. This is of course at least in part due to reverse causality: distrusting the news media leads people to seek alternatives [24]. However, this correlation is strongest among Republicans who spend more time driving and thus encounter a larger dose of talk radio, a result more consistent with effects of talk radio use on media trust than the reverse (see chapter 5 in [10]).

Particularly relevant here are studies that tested effects of bias accusations alongside the effects of actual favorability of news toward each party. A time series study by Watts and colleagues [8] found that accusations of media bias predicted increases in perceived media bias, but actual favorability of news for each party did not. The same results were found in one of the experiments cited above: elite media criticism reduced media trust, but the favorability of news coverage toward each party had no effect (see chapter 5, 2^nd^ experiment in [10]). These findings suggest attitudes about news are shaped by what others say about news, not direct observations of news content. In persuasion research, although individual messages rarely have strong effects, an imbalanced flow of arguments can accumulate into strong persuasive effects over time [25]. Thus, when messages attacking journalism are common and messages defending it are rare, trust is likely to erode no matter how good a job the news is doing.

Media literacy experiments provide the closest thing in past research to tests of effects of defending journalism, and have found that media literacy interventions can reduce perceived media bias [26][27][28]. These interventions, either in the form of public service announcements or classroom lessons, focus in part on explaining the professional norms and practices that aim to ensure impartiality of journalism [29][30]. This is not their only focus; they also address themes such as the importance of paying attention to sources the critical consumption of information. Thus, these experiments offer indirect evidence suggesting possible effects of defense of journalism.

### Effects of fact checking

Fact checking is a crucial function of journalism in a healthy democracy because of its theoretical potential to hold elites accountable and keep the national debate grounded in shared facts [2]. However, audience members do not always see fact checking as good journalism, as evidenced by backfire effects in which a fact check strengthens misperceptions or increases support for a politician caught in a lie [4][5][6]. Although these backfire effects do sometimes occur, research more often finds positive effects of fact checking [31][32][33][34][35][36][37]. Nevertheless, the possibility of backfiring warrants caution about assuming that audience members will always see fact checking as a sign of good journalism. Most fact checking experiments focus on correcting specific factual misperceptions; we focus on more general attitudes that may accumulate from fact checking across a diverse set of timely fact checks. A few studies have reported effects of fact checking on such general outcomes [33][35].

In particular, we examine epistemic political efficacy (EPE), which is self-confidence in an ability to figure out the accuracy of factual political claims [12]. Because EPE is about understanding politics, it is a better predictor of information seeking behaviors than traditional measures of internal or external efficacy that focus on confidence in taking effective political action [12][38][39]. Low levels of EPE are worrisome not only because they may lead some to tune out of political information seeking, but also because they may lead others to accept dishonest politics [12]. Three experiments have found that EPE can be influenced by fact checking within ordinary news stories [12][35][38]. When interest in a fact check topic is moderate to high, fact checking increases EPE [12][38], but when interest is very low it can actually decrease it [12][35]. All three of these experiments were limited by their use of a forced exposure experimental approach typical in media effects research, with participants assigned to read a news article they may not have chosen to read for themselves. In choice-driven real news use, presumably people will read more stories they are interested in, so fact checking should increase EPE.

## Hypotheses

As discussed above, we expect fact-checking effects to depend on whether the audience sees fact checking as a sign of good journalism or reacts against it as a sign of bias. We expect the defense of journalism manipulation to shift people from the latter interpretation to the former, resulting in a more positive effect of fact checking on all three outcomes than without defense of journalism. Thus, our main hypothesis is an interaction between fact checking and defense of journalism, such that defense of journalism makes the effects of fact checking more positive.

*H1: The effects of fact checking will be more positive with defense of journalism than without it, on a.) media trust, b.) epistemic political efficacy, and c.) future news use intent*.

We are also interested in two more basic questions: whether fact checking is effective with defense of journalism, and whether it is effective without defense of journalism. Effects of fact checking without defense of journalism are closer to the status quo in real news use, and correspond to past research on fact checking effects. As reviewed above, past research more often finds positive effects of fact checking. Accordingly, we hypothesize fact checking will have positive effects on all three outcomes both with and without defense of journalism.

*H2: With defense of journalism, fact checking will increase a.) media trust, b.) epistemic political efficacy, and c.) future news use intent*.

*H3: Without defense of journalism, fact checking will increase a.) media trust, b.) epistemic political efficacy, and c.) future news use intent*.

Because Democrats and Republicans differ greatly in media trust, and because of the partisan nature of media bias accusations as discussed above, it is important to examine whether any of the above effects are dependent on respondent party identity. It is plausible that only Democrats might be influenced by fact checking, by defense of journalism, or by their combination, or even that Democrats and Republicans might respond in opposite directions. Accordingly, we pose a research question about whether party identity interacts with either main effect or with their interaction.

*RQ: Does party identity moderate any effects of fact checking, defense of journalism, or their interaction, on a.) media trust, b.) epistemic political efficacy, or c.) future news use intent*?

## Methods

**Fig 1:**
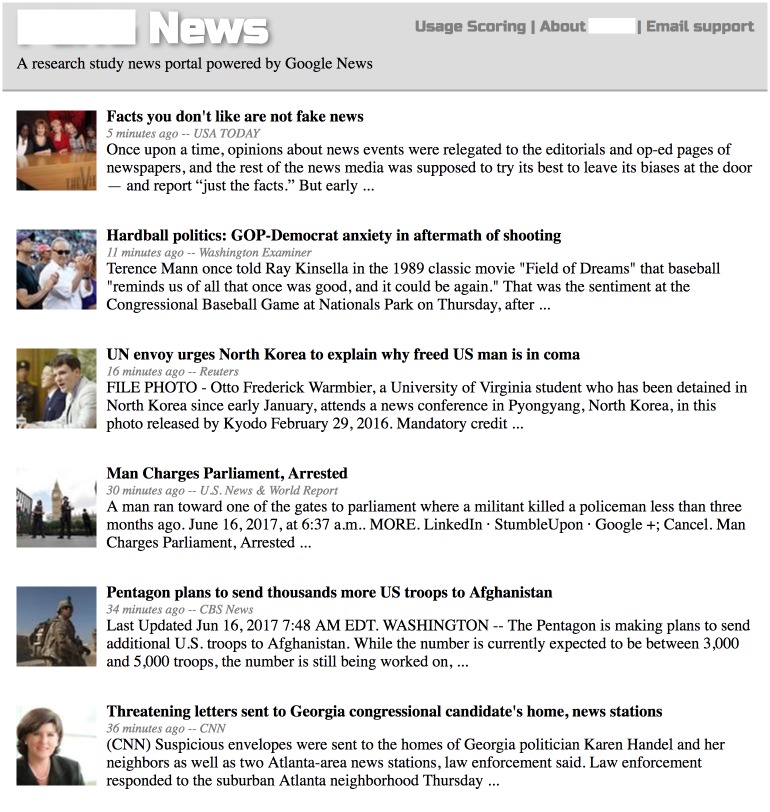
News portal screenshot.

A custom news portal website (see Fig 1) was created for this study and used by 1187 paid study participants during five days in June 2017. The portal was automatically updated with timely news stories from Google News at the top of every hour and included a total of 1196 stories. Stories were listed reverse-chronologically, and users could scroll down to automatically reveal older stories. Participants were free to choose when to visit the portal and which stories to read. Story categories were tentatively identified using keywords and then verified by researchers. Experimental factors added in stories based on these categories.

The experimental design used here was fully factorial 2 (fact checking) x 2 (defense of journalism). This was part of a broader fully factorial design that also included three other two-level factors intended for unrelated purposes (coverage of the Russia investigation, intra-party disagreement, and relative emphasis on health care versus immigration). A given story could be categorized in more than one of these categories. In such cases any one of the experimental factors could cause a story to be filtered out. Non-hypothesized factors were included in all analyses.

### Participants and procedures

A convenience sample of U.S. adults (final N = 1187) was recruited using Amazon’s Mechanical Turk service. The study was approved by the Louisiana State University Institutional Review Board on May 10, 2017, approval #E10487. Participants were paid to take a pre-test survey (N = 1830), use our custom news portal during five weekdays, then take a post-test survey (N = 1187). Participants were required to be U.S. residents, and were 55% female, 80% Caucasian, 58% with a college degree, 24% Republican, 40% Democrat, and 32% Independent, with an average age of 39 years (SD = 12.2). The news portal opened at 5am on Monday June 12, 2017 and closed at 10am Friday June 16, 2017. The post-test survey was available between 1 and 36 hours after the portal closed. On average, participants saw the headline and brief description of 268 stories (SD = 183.9) in the news portal (as shown in Fig 1), and clicked on an average of 27 of them to read in more detail (SD = 24.6).

Participants were paid $1 for each survey and up to an additional $3 based on how much they used the portal. These incentives were designed to produce a sample of regular online news users. As we explained to prospective participants and in reminder messages to participants, our modest bonuses for portal usage were not intended as compensation for spending more time reading news than they otherwise would. Instead, they were intended as compensation for using our news portal instead of other news sources. With the exception of future news use intent (detailed below), all measures used here were measured in both the pre-test and post-test. This is important here because the long duration of the study makes study dropout a concern. Controlling for pre-test measures of an outcome addresses the possibility that treatment group differences are explained by dropout instead of by within-subjects change.

### News story discovery and categorization

At the top of each hour, our PHP script used RSS queries to find new stories from Google News. A query for the top 15 stories in the Top Stories section of Google News was the baseline news feed. All stories in this feed were available to all participants unless an experimental treatment filtered them out. Out of a total of 1196 stories included in the portal, 1018 came from this baseline feed. Stories in the baseline feed were given a random publication delay between 5 and 60 minutes, to allow researchers to log in once per hour to verify categorization of stories before publication. This baseline feed was then supplemented by several keyword-based RSS queries, also all done at the top of each hour. These queries were used to cast a wide net for stories that might be used for experimental conditions, after verification by a researcher.

Researchers manually injected stories found from keyword feeds for each manipulation, selecting these stories based on their appropriateness for the intended manipulation, their newsworthiness, and their timeliness. Although timely stories were preferred, researchers could add non-timely stories as long as those stories did not contain obvious signs of age in their headline or introductory paragraph. The actual original publication date of each story was not shown in our portal (but could be found by clicking through to the actual story). Instead, visible publication dates and times were when each story first became visible in the portal. Researchers scheduled stories to be added for each manipulation several hours ahead of time to ensure regularity of coverage for each manipulation, then sometimes cancelled or delayed those scheduled stories if more timely options became available.

Candidate stories for the fact checking manipulation were found using an RSS query from Google News with “fact check” as the search term. This query turned up a total of 154 stories. 23 of these were manually added to the portal and constituted the fact checking manipulation (see S1 File). On average, participants in the fact checking condition saw 3.84 fact check headlines (SD = 2.98) and clicked on .54 of them (SD = .93). We cast a wide net for potential stories for the defense of journalism manipulation using an RSS query from Google News for stories matching any of the keywords “journalism,” “fake news,” or “bias.” This query turned up a total of 142 stories, only 12 of which were actually stories defending journalism. All 12 of these were used in the portal for the defense of journalism manipulation (see S1 File). Because so few defense of journalism stories were available, we published them in the portal largely at high usage times. On average, participants in the defense of journalism condition saw 3.71 defense of journalism headlines (SD = 2.75) and clicked on .58 of them (SD = 1.07). Note that although such headline exposure variables could conceivably be treated as mediators of experimental effects, extremely skewed distributions make such analyses problematic. For simplicity we rely instead on conventional experimental analyses.

### Measures

Survey items for media trust and EPE used 7-point scales with endpoints labeled “Strongly disagree” and “Strongly agree.” Media trust (pre-test M = 3.29, SD = 1.56, Cronbach’s alpha = .95; post-test M = 3.45, SD = 1.54, Cronbach’s alpha = .96) was an average of five items, each beginning with the phrase “In general, mainstream media outlets are,” and ending with “fair,” “accurate,” “unbiased,” “tell the whole story,” and “can be trusted.” Epistemic political efficacy (pre test M = 5.00, SD = 1.26, Cronbach’s alpha = .91; post-test M = 5.07, SD = 1.22, Cronbach’s alpha = .92) used the four-item scale from [38], with items such as “I feel confident that I can find the truth about political issues.” Future news use intent (post-test M = 5.15, SD = 1.65) was a single item measured on a 7-point likelihood scale from “Very unlikely” to “Very likely.” The item was “Use Google News or another similar service that includes news from many sources.”

## Results

For each outcome, three analysis of covariance (ANCOVA) models were used: A combined model including all participants tested H1, and two separate models including only participants in the defense of journalism or in the no defense of journalism condition were used to test H2 and H3, respectively. Each model included a two-level factor for the fact checking manipulation, a three level party identity factor (Democrat, Republican, or Independent / Other), the three non-hypothesized factors as main effects, and a two-way interaction between party identity and fact checking. The combined models also included the two-level factor for the defense of journalism manipulation, a two-way interaction between it and party identity, a two-way interaction between it and fact checking, and a three-way interaction between defense of journalism, party identity, and fact checking. For the first two outcomes, media trust and EPE, all models included a pre-test measure of the outcome variable as a covariate. The models predicting future news use intent did not include such a covariate because this outcome was not measured in the pre-test. Instead, these models controlled for pre-treatment portal usage, a logged measure of portal usage during an initial 24-hour period of the news portal before experimental treatments were activated.

**Fig 2:**
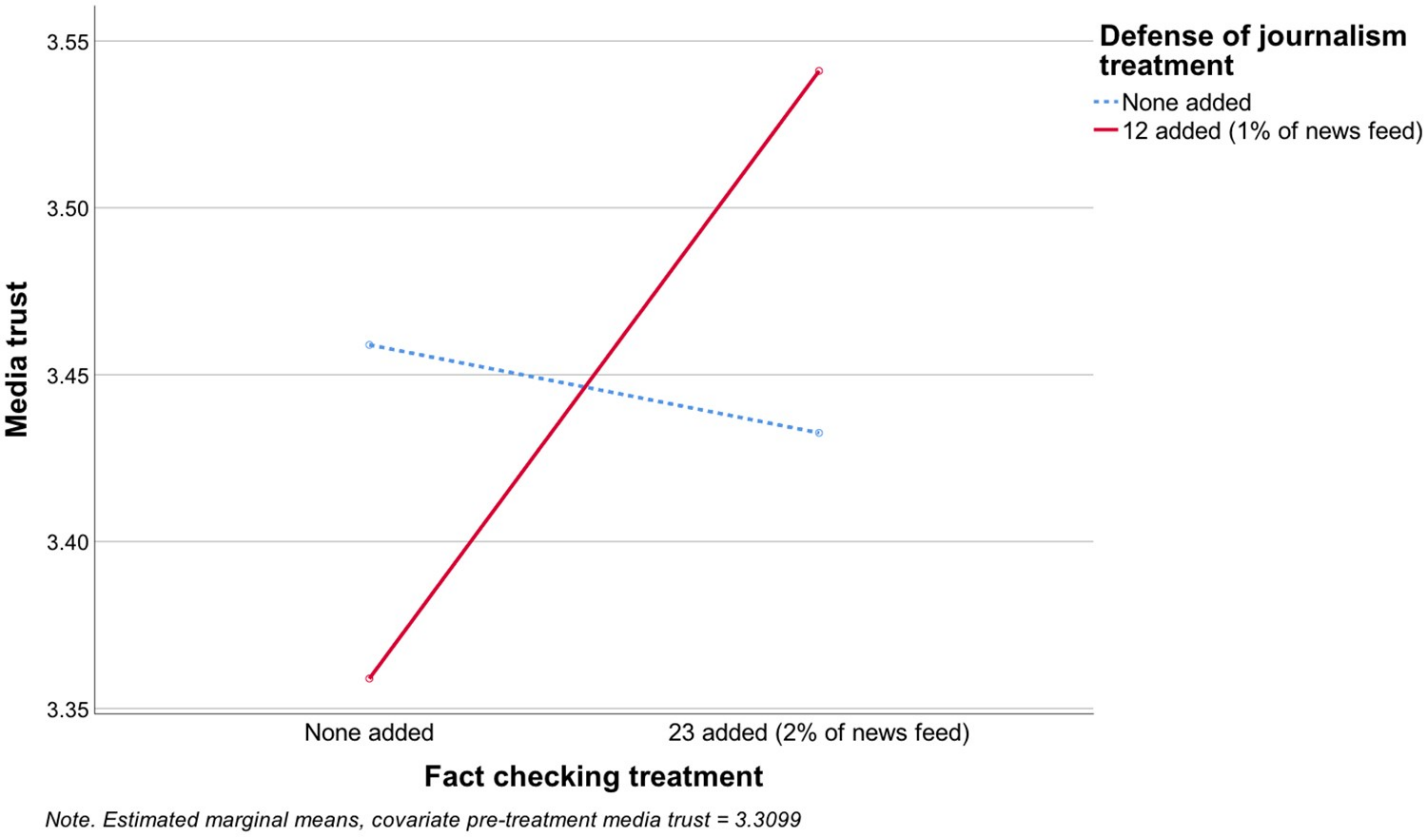
Effects on media trust.

In the combined model predicting media trust (adjusted R^2^ = .775), neither experimental factor had a significant main effect, but the interaction term for fact checking and defense of journalism was significant (F[1, 1110] = 5.45, p = .020), and in the predicted direction (see Fig 2), so H1a was supported. Among participants in the defense of journalism condition (adjusted R^2^ = .793), fact checking increased media trust (F[1, 543] = 8.290, p = .004), supporting H2a. Among participants in the no defense of journalism condition (adjusted R^2^ = .757), fact checking had no effect on media trust (F[1, 563] = .175, p = .676), so H3a was not supported.

**Fig 3:**
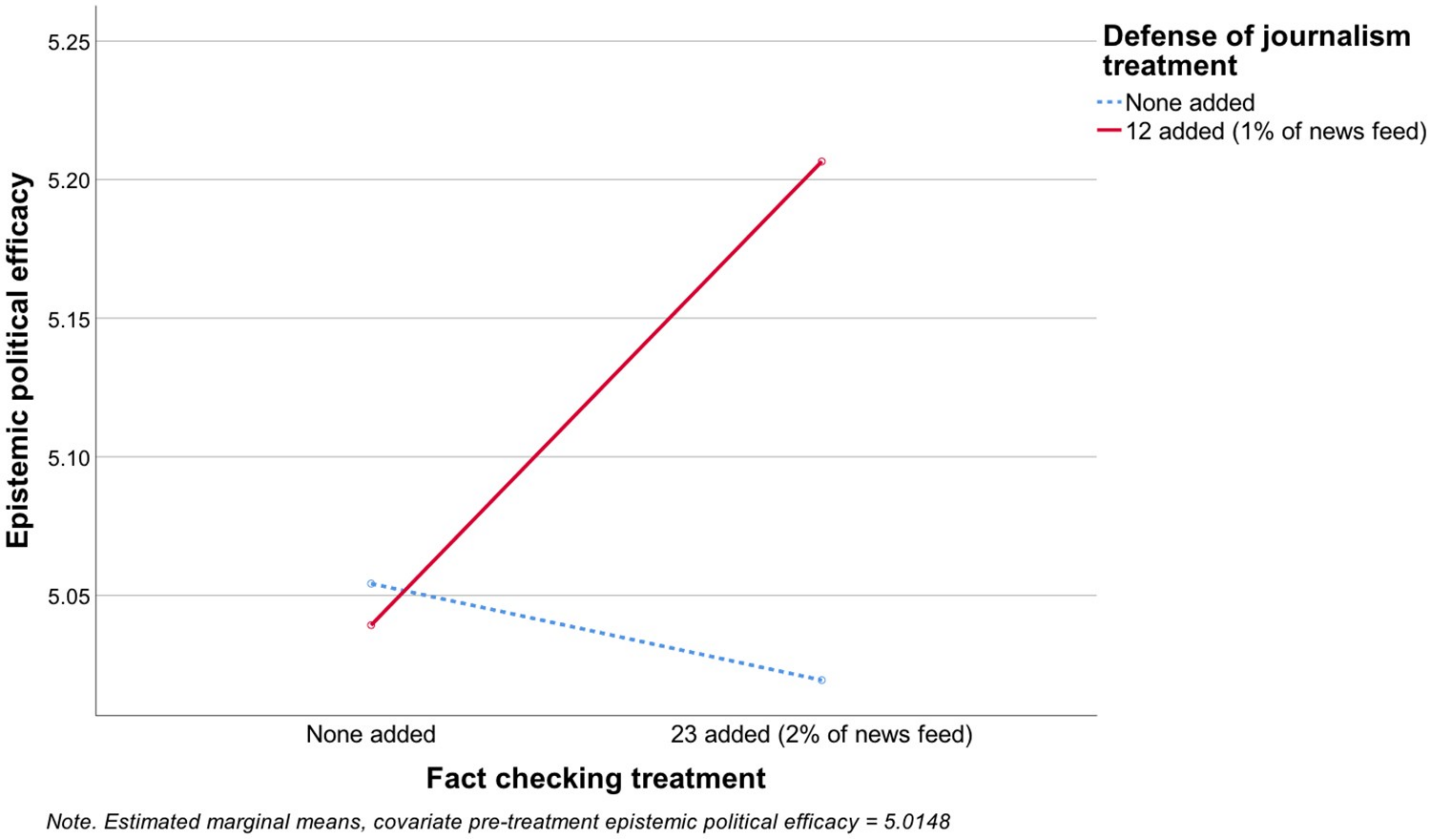
Effects on epistemic political efficacy.

The results for EPE mirror those for media trust. In the combined model predicting EPE (adjusted R^2^ = .559), neither experimental factor had a significant main effect, but the interaction term between fact checking and defense of journalism was significant (F[1, 1108] = 4.18, p = .041), and in the predicted direction (see Fig 3), so H1b was supported. Among participants in the defense of journalism condition (adjusted R^2^ = .542), fact checking increased EPE (F[1, 541] = 5.565, p = .019), supporting H2b. Among participants in the no defense of journalism condition (adjusted R^2^ = .573), fact checking had no effect on EPE (F[1, 563] = .331, p = .565), so H3b was not supported.

**Fig 4:**
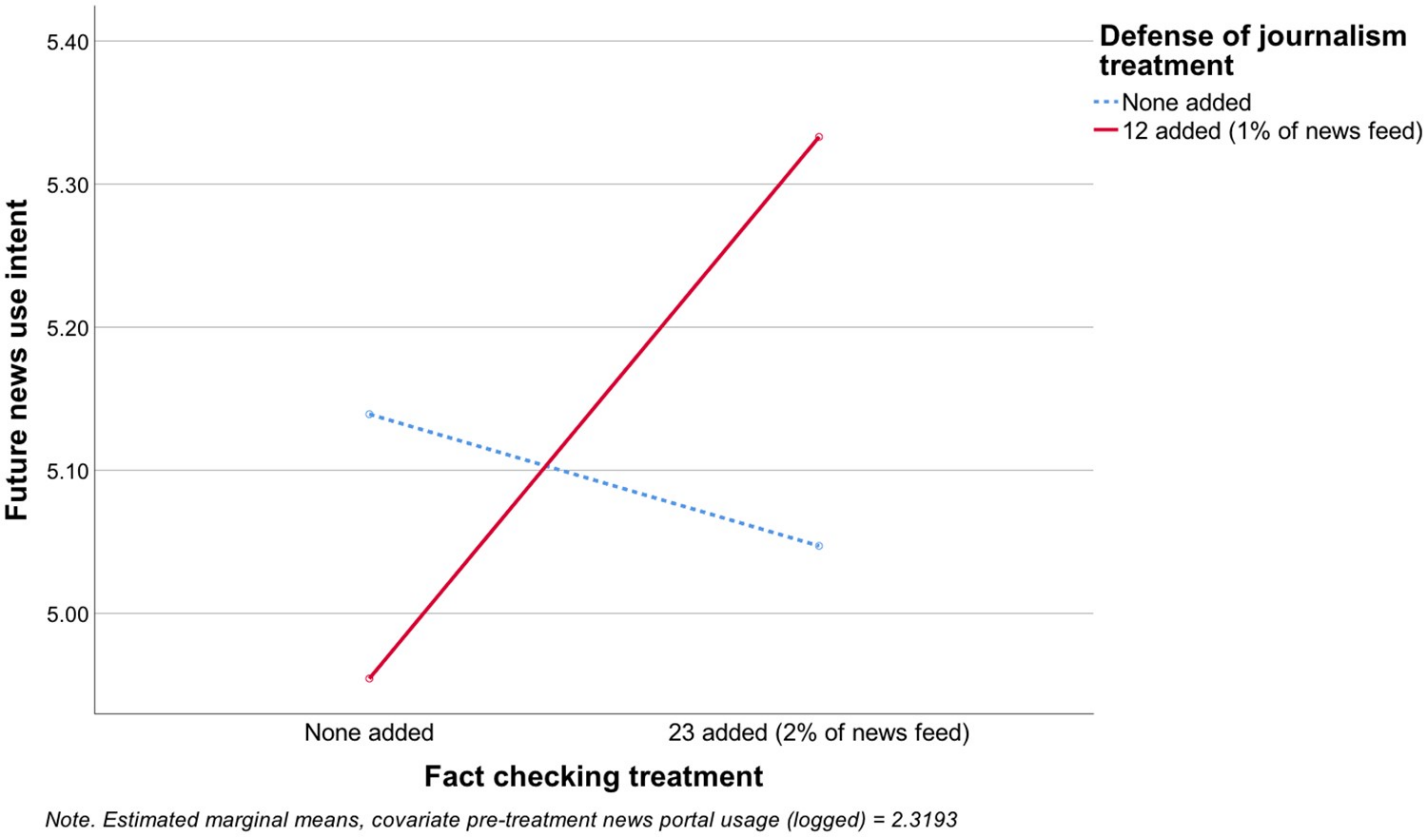
Effects on future news use intent.

The same pattern was also found for future news use intent. In the combined model predicting future news use intent (adjusted R^2^ = .026), neither experimental factor had a significant main effect, but the interaction term between fact checking and defense of journalism was significant (F[1, 1099] = 5.46, p = .020), and in the predicted direction (see Fig 4), so H1c was supported. Among participants in the defense of journalism condition (adjusted R^2^ = .019), fact checking increased future news use intent (F[1, 535] = 7.024, p = .008), supporting H2c. Among participants in the no defense of journalism condition (adjusted R^2^ = .031), fact checking had no effect on future news use intent (F[1, 560] = .364, p = .547), so H3c was not supported.

In answer to our research question, party identity did not interact significantly with the experimental main effects or with their interaction in predicting any of the outcomes. In the combined model predicting media trust, party identity did not interact significantly with defense of journalism (F[2, 1110] = 2.04, p = .131), fact checking (F[2, 1110] = 1.06, p = .348), or with their interaction (F[2, 1110] = .173, p = .841). In the combined model predicting EPE, party identity did not interact significantly with defense of journalism (F[2, 1108] = .094, p = .910), fact checking (F[2, 1108] = .327, p = .721), or with their interaction (F[2, 1108] = .022, p = .978). In the combined model predicting future news use intent, party identity did not interact significantly with defense of journalism (F[2, 1099] = 1.010, p = .364), fact checking (F[2, 1099] = 2.555, p = .078), or with their interaction (F[2, 1099] = .617, p = .540).

## Discussion

This study used a field experiment to test effects of defending journalism and fact checking on the audience during real online news use. We found positive effects of fact checking only when combined with stories that defended journalism. When a few opinion pieces defending journalism were included in an online news portal, fact checking increased participants’ trust in mainstream media, self-confidence in their own ability to decide what is true in politics, and intention to use a mainstream news portal in the future. Without defense of journalism, fact checking had no effect on any of these outcomes. Further, we did not find that party identity mattered at all in these effects. Although Republicans started out lower than Democrats on the three outcome variables, they were not significantly different in how much they were affected by fact checking, defense of journalism, or their combination.

Although the effects found here were fairly small, they resulted from a very small intervention of a handful of stories during a single week of news use. Because we used repeated exposure to stimuli and a one-hour delay between the last possible stimulus exposure and the first possible measurement of effects, these effects represent an accumulation of effects over time, and cannot be explained as fleeting effects driven by temporary cognitive accessibility. Thus, it is reasonable to assume these effects would continue to accumulate into larger effects over longer periods, but this should be verified by future research.

Perhaps the most important limitation of this study is that it does not incorporate how elites respond to defense of journalism. It is possible that the positive effects of defense of journalism found here would not generalize to the real world because defense of journalism would lead to additional anti-media rhetoric. Alternatively, one might expect our effects to generalize despite elite responses either because anti-media rhetoric may already be at a ceiling effect where additional exposure doesn’t have additional effects, or because no matter what journalists do this rhetoric will occur. Another possible outcome is a response by political elites on both sides, with both pro- and anti-media rhetoric, which might simply cancel each other out, or might instead make partisanship a strong moderator of effects, as we expected but did not find in this study. Future research conducted at times when defense of journalism is much more frequent and prominent could address these possibilities by incorporating exposure to elite rhetoric about media.

This study employed an innovative field experiment approach to studying effects of online news. This method combines strengths of observational studies of real news use with some of the strengths of a laboratory experiment. Because participants were randomly assigned to conditions, we can draw strong causal conclusions. Because we used real, timely stories as experimental stimuli embedded among other timely stories, and because we allowed participants to choose which stories to read, we can make stronger claims that the results correspond to what occurs in everyday online news use. However, this substantial improvement in realism comes at a cost of uncertainty about the active ingredient within a complex stimulus. For instance, it is possible that something within our 23 fact check stories other than the actual fact checking–say, coverage of a specific issue–explains effects of this manipulation. Future research should replicate these results in laboratory experiments where the inclusion of fact checking or defense of journalism within a stimulus news story can be isolated from other differences. Fact checking is of course important in ordinary news stories, not just in specialized fact check segments. Similarly, our defense of journalism manipulation used opinion pieces, but ordinary news stories that quote elites attacking the media could perhaps be balanced by also including quotes from a journalist defending the profession. Another important limitation is that due to a non-representative sample, these effects may not generalize to the population. This concern is lessened somewhat because we do not aim to accurately estimate population means but instead to estimate changes in means in response to manipulations. Nevertheless, it is important for future research to attempt to replicate these results with a representative sample.

Although we prefer the term field experiment for our method, some might argue that a true field experiment of news effects requires collaboration with a news outlet willing to randomly assign its real audience members to different versions of its coverage. Several such experiments have been done, but they remain rare because of the difficulty and constraints imposed by this collaboration, including unwillingness to enact certain manipulations and the necessity in old media systems of geographically-based clustered random assignment instead of individual random assignment [40]. Our experiment asks participants to do one artificial thing: switch to our news portal instead of their usual news sources. After this, the content within our portal is then all real, timely news. In our view, this allows it to effectively function as a field experiment without the difficulties arising from collaboration with a news organization. To our knowledge, other than our own work, there has been only one other study (currently in press) using a news portal field experiment similar to ours: a three-month study of learning from hard versus soft news in a news portal in Japan [41]. We are not aware of any past field experiment that has addressed effects of either fact checking or defense of journalism.

Particularly striking in relation to past fact checking research is the lack of differences in effects between Republicans, Democrats, and Independents. Republicans were lowest on all three outcomes, but did not significantly differ from Democrats or from Independents in how they changed in response to the stimuli. This might suggest that reactance against fact checking sometimes observed in past research was an artifact of laboratory experiment settings in which participants did not choose to read the fact check stimuli for themselves. However, our results don’t directly contradict past research because we don’t use stimulus-specific outcome variables such as factual beliefs. Because we manipulated a diverse set of timely fact check stories, we used general outcome variables such as media trust. Our results suggest media trust deserves more attention in fact checking research, and not just as an outcome. Low trust in mainstream media may help explain past backfire effects on these stimulus-specific outcomes. If so, our results would also suggest that the combination of fact checking with defense of journalism could make fact checking more effective not just in terms of the general outcomes studied here, but also in terms of increased correctness of factual beliefs and increased reputational costs for dishonesty by political elites.

The effects on EPE found here do differ from past research, but in view of methodological differences we do not see them as contradictory. Our participants were free to choose which stories to read, so they presumably read fewer fact-checking stories on low interest topics. Given this methodological difference, positive main effects of fact checking on EPE would be a reasonable expectation based on past research, which found negative effects only when people were experimentally assigned to read stories on topics they were uninterested in [12][35]. We found such positive effects, but only in the defense of journalism condition. This could be due to a difference in attitudes toward media in the adult convenience sample used here, compared to the undergraduate student convenience samples in past EPE experiments drawn from mass communication classes. Presumably many of those undergraduates received defense of journalism in mass communication classes, which could explain why they responded more similarly to those in our defense of journalism condition.

## Conclusions

These findings offer new hope for restoring trust in and use of news, as well as trust in the very notion of political facts. Others have concluded that restoring media trust would require an unlikely and undesirable return to the media landscape of the 1950s and 1960s, when a few outlets dominated and media trust was high [10]. This is an understandable position in view of the role of media fragmentation in allowing the rise of partisan outlets rich in anti-media rhetoric. What this misses is the potential role of past unwillingness of the press to respond to attacks, creating an unbalanced flow of arguments about the media.

Our results also bring new data to a long-standing debate among journalists. Although empirical data can’t contradict the principle that journalists should remain neutral when attacked, it can and does contradict a claim often used in support of this principle. At least in terms of the outcomes studied here, our results contradict the claim that the most effective response is to ignore anti-media rhetoric and just do good journalism. This is consistent with past research finding that media trust is shaped by what others say about the news media, not by direct observation of news content [8][10]. Due to the alarming extremity of recent anti-media rhetoric, at least some journalists are beginning to respond. However, it seems to us that prominent defense of journalism by major outlets occurs mostly when anti-media rhetoric reaches new extremes, such as when the President began calling the mainstream press fake news or the enemy of the American people. Subsequent repetitions of this same rhetoric do not seem to have produced much defense of journalism in response, but this should be assessed empirically with content analysis.

We suggest the line should be drawn much sooner, at any accusations of *intentional* bias. Mainstream journalism needs to be defended not only against claims that it is equivalent to fake news, but also against claims that it is equivalent to partisan news. This does not require arguing that unintentional bias never occurs or that mistakes are never made. It simply requires insisting that the press is trying to be the impartial referee of political facts that democracy needs it to be. A misperception that all news is partisan news for one side or the other undermines the ability of the press to fulfill its essential role in democracy. We also do not think it is wise for journalists to rely on any other actor in society to persuasively make this argument. Journalists are the only ones who can speak authoritatively about their own intentions, professional norms, and organizational safeguards against bias. They may continue to choose not to defend their profession out of adherence to a principle of neutrality, but our results suggest they should not do so on the assumption that the public will see this neutrality as proof that the critics are wrong.

These results suggest that this combination of defense of journalism with fact checking could help reverse these alarming trends, restoring trust in and use of mainstream news while restoring faith in the attainability of political facts. Much more research is needed to replicate these results in other contexts and try variations of these interventions, such as defense of journalism from various sources, explanation of journalism instead of defense (journalists may be more comfortable with this), social media news feeds instead of a news portal, fact checking and defense of journalism embedded within ordinary news stories, and both longer- and shorter-term interventions. We hope other researchers will join us in testing these possibilities with a variety of methods.

## Supporting information

S1 File Experimental treatment stories. (PDF)
