# Supplementary material for: Checking facts and fighting back: Why journalists should defend their profession
Source: PLoS One. 2018 Dec 10;13(12):e0208600. doi: 10.1371/journal.pone.0208600 (PMC6287821; doi:10.1371/journal.pone.0208600)
Supplement: S1 File — (PDF) [file pone.0208600.s001.pdf]

Defense of journalism treatment stories

National Review 5/17/17	How to Read the Newspaper
The Nation 5/26/17	Conservatives Complain That Republicans Have a Liberal bias
Romper 5/28/17	Trump Accused The Media Of Making Up Sources
Valley Advocate 5/30/17	Working Against Bad Political Actors Is Not Bias — It's Journalism
HuffPost 5/30/17	Defending Journalism In The Age of Trump
Newsmax 6/5/17	Newseum Boss: Journalism in Jeopardy Under Trump
USA Today 6/7/17	Facts you don't like are not fake news
WIRED 6/8/17	In a Fake Fact Era, Schools Teach the ABCs of News
New York Post 6/12/17	Muehller's conflict of interest, the price of assassinating 'Trump' and other comments
The Gazette 6/12/17	GOP strategists plot anti-media strategy for 2018
Slate Magazine 6/13/17	Fox News, Filter Bubbles, and Fake News
The Conversation US 6/16/17	How a journalism class is teaching middle schoolers to fight fake news

Fact checking treatment stories

ABC News 6/6/17	AP FACT CHECK: Trump misdirects blame for slow confirmations
Snopes 6/6/17	Did London Mayor Sadiq Khan Defend 9/11 Terrorists?
PolitiFact 6/7/17	Fact-check: Handel claims Obama flew $2 billion to Iran, admitted it is being used to fund terrorism
The Daily Caller 6/7/17	FACT CHECK: Have Small Banks Struggled Under Dodd-Frank?
USA TODAY 6/9/17	Fact check: The Comey hearing
Snopes 6/9/17	Imelda Marcos Death Hoax
Washington Post 6/9/17	Study: Conservatives despise the fact-checking industry
The Detroit News 6/9/17	Fact check: Trump says Comey cleared him. He didn't
ABC News 6/10/17	AP FACT CHECK: Assessing Trump's self-defense in Comey drama
Fort Wayne Journal Gazette 6/12/17	Fact check: Planned Parenthood's claim about birth control access for "99% of sexually active women"
Chicago Tribune 6/12/17	Fact check: The record behind Trump's Cabinet meeting declarations
Washington Post 6/13/17	President Trump's claim his nominees faced "record-setting long" delays
Minneapolis Star Tribune	AP FACT CHECK: Trump appointees see quicker ethics

6/13/17	process
Post-Bulletin 6/13/17	AP FACT CHECK: Pence's Obamacare "death spiral" chart
Florida Times-Union 6/13/17	Fact Check: Celebrities aren't threatening to strike over Trump
Haaretz 6/14/17	Fact Check: Is Qatar Supporting Terrorism? A Look at Its Ties to Iran, ISIS and the Muslim Brotherhood
The Daily Caller 6/14/17	FACT CHECK: Half The People In Louisiana Jails Haven't Been Convicted Of A Crime
People's Pundit Daily 6/14/17	Fact-Checking Terry McAuliffe's "93 Million" Lost to Gun Violence Claim
Lifesite 6/14/17	The liberal media is pretending abortion doesn't cause breast cancer
Washington Post 6/15/17	President Trump's mangled "facts" about Obamacare
Snopes 6/15/17	Rand Paul Tweeted That Purpose of Second Amendment Is to Shoot at Tyrannical Government?
Snopes 6/15/17	Was a Man Accused of Ejaculating in His Boss's Coffee Daily for Four Years?
Philly.com 6/15/17	Factcheck: Pre-existing condition spin
